# Jejunal Gastrointestinal Stromal Tumor (GIST) as a Rare Cause of GI Bleed: A Case Report

**DOI:** 10.7759/cureus.24272

**Published:** 2022-04-19

**Authors:** Haoming Liu, Abbey Santanello, Mercy Jimenez, Narendra Kumthekar

**Affiliations:** 1 Medicine, Touro College of Osteopathic Medicine, Middletown, USA; 2 Medicine, Touro College of Osteopathic Medicine, New York City, USA; 3 Surgery, Flushing Hospital Medical Center, Flushing, USA; 4 Surgery, Kingsbrook Jewish Medical Center, Brooklyn, USA

**Keywords:** upper gastrointestinal bleed, gastrointestinal stromal tumor (gist), jejunum, abscess, tumor imaging, laparascopic surgery

## Abstract

Jejunal gastrointestinal stromal tumor (GIST) is a rare cause of recurrent gastrointestinal bleeding (GIB). Early diagnosis for patients with jejunal GIST is often challenging, which can lead to delays in treatment. We present a case of a 32-year-old male patient with persistent abdominal pain and hematemesis despite treatment for gastroesophageal reflux disease (GERD). Upon initial ER visit, CT result was consistent with intra-abdominal abscess and the patient underwent interventional radiology (IR) drainage. On a second ER visit three weeks later, CT showed a suspicious lesion in the small bowel. The patient underwent exploratory laparoscopy which revealed a mass in the jejunum. The lesion was resected successfully and pathology report confirmed the diagnosis of GIST with positive immunohistochemistry marker cluster of differentiation (CD)117. The patient was discharged with no complications post-operatively. In conclusion, recurrent GIB and unusual imaging findings should raise clinical suspicion for alternative causes for GIB, including tumors such as GIST.

## Introduction

Gastrointestinal stromal tumors (GISTs) are rare tumors of the gastrointestinal tract of mesenchymal origin. Presenting symptoms can include nausea, vomiting, and abdominal fullness, though approximately 30% of cases are asymptomatic [1]. GIST represents 1% of all gastrointestinal neoplasms [1,2]. The most common location in the gastrointestinal tract is the stomach, followed by the small intestine, and only 10% originate in the jejunum [3]. GISTs arise from the interstitial cells of Cajal. These cells function to generate electrical slow-wave activity, coordinate pacemaker activity, and transduce motor neural inputs from the enteric nervous system [4]. 

Due to the nonspecific nature of the symptoms and limitations of diagnostic imaging studies, it is challenging to confirm the diagnosis of jejunal GIST pre-operatively. Perforations are more common for GISTs of the small bowel compared to other anatomical sites [5]. CT is the first-line imaging tool for diagnosis [6]. MRI may be offered as an alternative if intravenous CT contrast is contraindicated [6]. Positron emission tomography (PET) scanning is useful for assessing metastatic diseases and determining the response to tyrosine kinase inhibitor therapy [7]. Pathology of the tumor can exhibit necrosis (18.8%) or mucosal ulceration (37.5%), which potentially indicates the cause of gastrointestinal bleeding [6]. The diagnosis of GIST is confirmed with pathologic evaluation and immunohistochemistry. The most prominent hallmark of GIST is receptor tyrosine kinase KIT, or cluster of differentiation (CD)117, which is specific to GIST and distinguishes it from other mesenchymal cell tumors, such as leiomyoma and leiomyosarcoma. Other markers such as DOG-1, CD-34, smooth muscle actin (SMA), S100, and Desmin are also occasionally positive [2]. 

Biopsy and excision are the preferred first-line treatment modality for GIST >2 cm as they are associated with a high risk of progressive disease [1]. Tyrosine kinase inhibitors, with the prototype imatinib, have been shown to increase survival as neoadjuvant and adjuvant therapy with specific indications [8,9].

## Case presentation

We report the case of a patient with hematemesis and recurrent abdominal pain who was initially diagnosed with an intra-abdominal abscess. Diagnostic laparoscopy and pathology eventually revealed a small bowel tumor and specifically, a GIST of the jejunum. 

**Figure 1:**
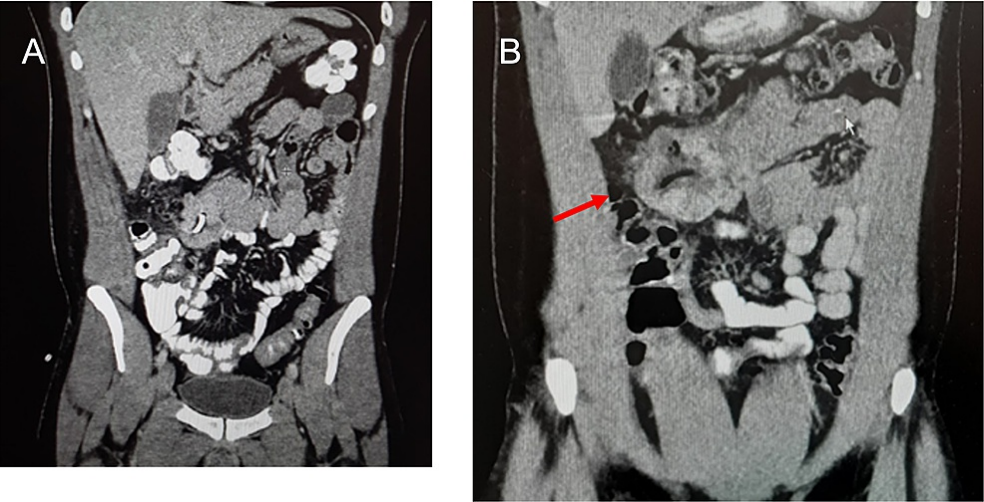
Side-by-side comparison of CT abdomen and pelvis three weeks apart. (A) Coronal CT scan after percutaneous drainage of the abscess was performed. (B) Coronal CT of the same patient three weeks later shows an abscess with complex features suggesting a mass of the small bowel. The size of the mass is 6 x 5.5 x 5 cm, similar to the abscess drained during the initial presentation.

The patient was a 32-year-old male who presented to the ER with one month of abdominal pain, nausea, vomiting, and hematemesis. He had been treated for GERD as an outpatient, however, proton pump inhibitors did not relieve the symptoms. He reported no prior medical conditions or surgical procedures. Social history including alcohol use was negative. There was also no family history of malignancy. In the ER, the patient was initially tachycardic with a heart rate in the 120s beats per minute (bpm) and hypotensive with BP averaging 90/60 mm Hg but responded to IV fluid boluses. Laboratory findings were significant for WBC count of 23.2 x 10^3^/mcL, hemoglobin of 11.0 g/dL, hematocrit (HCT) of 35%, and platelet count of 263 x 10^3^/mcL. Initial CT of the abdomen and pelvis with contrast demonstrated an area with the appearance of an intra-abdominal mesenteric abscess, which measured 6.9 cm in greatest transverse diameter. The patient was admitted for IV antibiotics and underwent CT-guided percutaneous drainage of the abscess and a pigtail catheter was left in place. Repeat CT demonstrated a significant reduction of fluid and air within the abscess contents (Figure 1, panel A). The patient was discharged with the drain and antibiotics when tolerating a diet and symptoms had resolved.

There was no recorded follow-up until the patient then returned to the ER with recurrent abdominal pain three weeks later. On examination, the abdomen was soft with tenderness to palpation in the right upper quadrant and epigastrium. A palpable mass was present in the right upper quadrant. There was no resolution of symptoms with continued IV antibiotics and there was minimal drainage from the pigtail catheter. Repeat hemoglobin on follow-up was 12.5 g/dL. Tumor markers for carcinoembryonic antigen (CEA), cancer antigen (CA) 15-3, serum alpha-fetoprotein (AFP), CA 125, and CA 19-9 were all within normal limits. Repeat CT scan of the abdomen demonstrated a persistent abscess with a thick-walled “shell” protruding from the wall of the small bowel (Figure 1, panel B). The abscess represented more complex features compared with previous imaging, suggesting hemorrhage and necrosis, and possible tumor growth. There was also trace-free fluid in the pelvis. The patient was taken to the operating room for a diagnostic laparoscopy and possible laparotomy.

**Figure 2:**
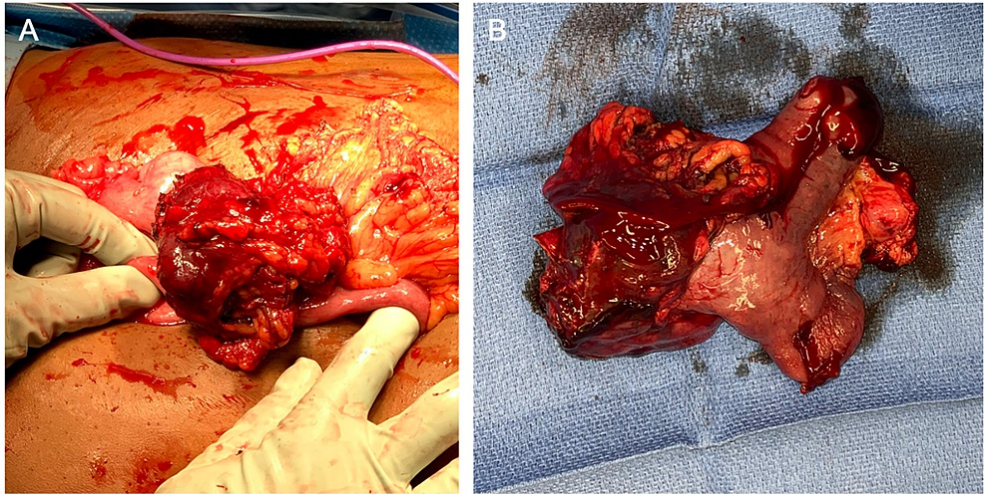
Laparotomy revealed mass in the jejunum. (A) At laparotomy - mass at distal jejunum with areas of necrosis and ulcerations on the wall of the mass. (B) The mass was resected with normal proximal and distal margins of the small bowel.

Intra-operatively, a large mass in the small bowel with a fistulous tract around the existing drain was detected. The procedure was converted to a laparotomy. The tract was excised along with a fungating mass in distal jejunum with a size of 6 x 5.5 x 5 cm (Figure 2, panels A and B). The mass was resected en bloc with normal proximal and distal margins while taking care to include mesentery down to the origin of the jejunoileal branch of the superior mesenteric artery. A functional end-to-end anastomosis was performed using a standard GIA stapler. The postoperative course was uncomplicated. Antibiotics were discontinued and the patient was subsequently discharged with instructions to follow up with primary care provider.

The resected mass was sent to pathology for further evaluation. Gross pathology demonstrated an ulcerated and necrotic tumor measuring 6 x 5 x 5 cm. Initial pathology report demonstrated a leiomyoma of the small bowel but further analysis showed that it was a GIST. The immunohistochemistry staining revealed CD117 positive, CD34 negative, SMA positive, desmin negative, S100 negative, and DOG1 positive. Ki67 <1%. These immunohistochemistry findings confirmed the diagnosis of a gastrointestinal stromal tumor of the jejunum. The tumor was found to have a mitotic activity of 1 mitosis/50 high-power fields (HPF). Progressive-free survival is 76% according to the study of pathology and prognosis of GIST at different sites by Miettinen and Lasota [10]. At this time, imatinib was not incorporated into the treatment plan, but the patient was encouraged to follow up for further discussion and recommendation.

## Discussion

Neoplasms of the upper GI tract account for 5% of cases of severe upper gastrointestinal bleeding, with most tumors being diagnosed at an advanced stage [11]. Although rare overall, it has been found that 83% of tumors originally classified as smooth muscle tumors of the jejunum and ileum were GISTs. These tumors varied from 0.3 to 40 cm with a median of 7.0 cm [10]. The estimated 15-year recurrence-free survival after surgery of GIST is 59.9% (95% CI: 56.2-63.6); the same mortality rate (39%) was found in age groups above and below 40 years [10,12]. 

The risk of GIST to develop progressive disease, as defined by metastasis and tumor-related death, depends on multiple variables including completeness of resection, tumor size, mitotic activity, location, and the presence of rupture [10]. A guideline of risk assessment has been proposed based on the long-term follow-up of gastric, small intestinal, duodenal, and rectal GISTs before the use of tyrosine kinase inhibitors (Table 1) [10]. Of note, duodenal GISTs are incredibly rare because of the difficulty to diagnose due to the complex anatomy. They are often separated from the data for small intestinal GISTs as they have a highly variable clinical presentation, typically smaller size (on average 4-5 cm), and are often diagnosed incidentally [10,13-15]. 

**Table 1:** Risk assessment of gastrointestinal stromal tumors (GISTs) in different locations. The required total count of mitoses is per 5 mm^2^ on the glass slide section; 50 HPF is equivalent to 5 mm^2^ on older microscopes, whereas most modern microscopes with wider 40x lenses require 20 HPF to embrace 5 mm^2^. Measurement of field of view may be necessary to accurately determine the number of fields required to count 5 mm^2^ on individual microscopes. *Metastasis or tumor-related death. **A small number of cases.

Tumor parameters		% Progressive disease*			
Mitotic count	Size (cm)	Gastric	Duodenum	Jejunum/ileum	Rectum
≤5/50 high-power fields (HPF)	<2	0	0	0	0
	2-5	1.9	8.3	4.3	8.5
	5-10	3.6	Insufficient data	24	Insufficient data
	>10	10	34	52	57
>5/50 HPF	<2	0	Insufficient data	High**	54
	2-5	16	50	73	52
	5-10	55	Insufficient data	85	Insufficient data
	>10	86	86	90	71

GISTs that are found in the muscularis propria of the jejunal wall have the potential to ulcerate and perforate [1]. In addition to obstructing the lumen of the small bowel or surrounding organs, perforation can cause severe bleeding and anemia. Tumor rupture, either spontaneously or intraoperatively, is an independent risk factor for disease progression and indicates high risk which requires adjuvant treatment with Imatinib [16-18].

Treating patients with GIST involves a multifactorial consideration based on risk of tumor progression, immunohistochemical staining, specimen margin, and mutational status [8,9]. Surgical resection is the definitive therapy to reduce the risk of malignant transformation and recurrence. Post-operatively, observation is all that is recommended if an R0 resection (negative microscopic margins) was achieved [8,19]. 

The gain-of-function mutations associated with GISTs are most commonly found in exon 11 (70% of total GISTs) or exon 9 (10% of total) of the KIT gene in the interstitial cells of Cajal. The second most commonly mutated gene is platelet-derived growth factor receptor alpha (PDGFRA). Therefore, tyrosine kinase inhibitors (TKI) that block the signaling of c-KIT tyrosine kinase at the PDGFRA receptor are recommended as therapy when indicated [8,9]. Imatinib is the prototype TKI, while sunitinib, ripretinib, and avapritinib have also been used with varying effects and specificity to the PDGFRA receptor. Post-operative imatinib treatment for approximately six to nine months is recommended if the operative specimen has positive microscopic margin [8]. TKIs are also used as neoadjuvant therapy for six to nine months and continued post-operatively for unresectable GISTs to reduce tumor size and decrease morbidity associated with surgical resection. In addition, TKIs are indicated as adjuvant therapy for GISTs with specific mutations (as certain mutations are associated with primary resistance to imatinib) and a high risk of recurrence or metastatic disease [9]. For example, individuals who harbor a KIT mutation-negative tumor with a D842V mutation in the PDGFRA gene, succinate dehydrogenase (SDH)-deficient GIST, or neurofibromatosis (NF)-related GIST should not be offered TKI therapy.

## Conclusions

Jejunal GISTs are incredibly rare, comprising 0.04% of all GI tumors, and may cause hemorrhage, rupture, and metastasis. It is important to keep jejunal GIST in mind when managing patients with hematemesis or a complex intra-abdominal abscess with an atypical presentation or unknown source, in order to prevent delays in treatment and severe outcomes. In addition, there exists a need for further understanding of the pathophysiology of GISTs and for diagnostic advancements to ensure optimal patient care.
